# Dietary intervention reduces left atrial enlargement in dogs with early preclinical myxomatous mitral valve disease: a blinded randomized controlled study in 36 dogs

**DOI:** 10.1186/s12917-019-2169-1

**Published:** 2019-11-27

**Authors:** Qinghong Li, Allison Heaney, Natalie Langenfeld-McCoy, Brittany Vester Boler, Dorothy P. Laflamme

**Affiliations:** 1 Nestlé Purina Research, St. Louis, MO USA; 2 Petcardia Veterinary Cardiology, Boulder, CO USA; 3 Scientific Communications Consultant, Floyd, VA USA

**Keywords:** Dietary intervention, Mitral valve disease, Dogs, Medium chain triglycerides, Congestive heart failure, Randomized controlled trial

## Abstract

**Background:**

Myxomatous mitral valve disease (MMVD), the most common naturally-occurring heart disease in dogs, is associated with alterations in energy metabolism, oxidative stress and inflammation. Energy deprivation plays a causal role in the development of heart failure. This study was designed to determine if a cardiac protection blend (CPB) of nutrients containing medium-chain triglycerides as an alternative energy source, fish oil to reduce inflammation, antioxidants, and other key nutrients important to cardiac health and function could slow or prevent MMVD progression. Nineteen dogs with early stage MMVD and 17 breed-, age-, and sex-matched healthy dogs were enrolled for a 6-month blinded, placebo-controlled study. Dogs in each cardiac health group were randomly assigned to either control diet (CON) or CPB-supplemented diet. Echocardiography was performed at baseline, 3 months and 6 months.

**Results:**

No changes were found in healthy dogs. While MMVD-CON dogs had an average 10% increase over baseline in left atrial diameter (LAD) and left atrial to aortic root ratio (LA/Ao) at 6 months, MMVD-CPB dogs showed 3% decreases, resulting significant diet by time interactions (*P* = 0.037, *P* = 0.005, respectively). More MMVD-CON dogs progressed from stage B1 to B2 during the study. A positive correlation was found between 6-month changes in LAD and blood pressures in MMVD-CPB dogs (systolic: *P* = 0.050, diastolic: *P* = 0.035) but not MMVD-CON dogs.

**Conclusions:**

Our results demonstrated efficacy of CPB-based dietary intervention in reducing LA size and mitral regurgitation, and in slowing or preventing the progression of early MMVD in dogs.

## Background

Canine myxomatous mitral valve disease (MMVD) is a common, naturally-occurring condition in dogs characterized by slowly progressive valvular degeneration that causes mitral regurgitation. According to the consensus statements from the American College of Veterinary Internal Medicine (ACVIM) [1], dogs in the preclinical stage with a heart murmur due to mitral regurgitation but without clinical signs of congestive heart failure (CHF) are classified as stage B, which is further classified into B1 or B2 as evidenced by the absence or presence of cardiac remodeling. Dogs with overt clinical signs of heart failure are classified as stage C. MMVD generally progresses slowly with a lengthy preclinical period. But once entering stage C with CHF, the disease advances more rapidly with a mean survival time less than 12 months [2]. Thus, it is of great interest to slow or prevent the progression of MMVD at the early preclinical stages to extend longevity and enhance quality of life for affected dogs.

Under normal conditions, approximately 70–90% of ATP generated in the adult mammalian heart comes from fatty acid oxidation, with the balance coming from oxidation of glucose, lactate and other energy substrates [3–5]. Significant evidence indicates that mitochondrial dysfunction leading to energy deprivation to the myocardium plays a causal role in the development of heart failure [6–10]. A shift to increased reliance on glycolysis as the main energy source in the context of reduced capacity in fatty acid oxidation has been reported in the failing heart in rodent models [11]. In addition, oxidative stress secondary to mitochondrial dysfunction or other causes, is strongly associated with cardiac diseases and failure [10, 12, 13].

Canine MMVD is associated with numerous metabolic changes that may be a cause or consequence of this disease. Previous metabolomic and transcriptomic research evaluating both cardiac tissues and serum samples documented a number of cellular and metabolic changes in dogs with MMVD [14]. Most of these changes could be collectively categorized as alterations in energy metabolism, oxidative stress, inflammation and extracellular matrix homeostasis pathways [14–16]. Markers of energy metabolism in MMVD dogs showed compromised fatty acid oxidation and ketosis, and increased reliance on anaerobic glucose metabolism. The objective of this study was to evaluate the clinical impact of a diet containing a nutrient blend designed to address these metabolic changes in dogs with naturally-occurring, early stage MMVD.

The 8- and 10-carbon fatty acids (Caprylic acid and Capric acid, respectively) from medium chain triglycerides (MCTs) provide a potential alternate energy source. MCTs are readily digested and absorbed, and the resulting fatty acids can cross the mitochondrial barrier without requirement for carnitine, and are rapidly oxidized. A MCT-supplemented diet has been shown to prevent progressive cardiac remodeling in spontaneously hypertensive rats, possibly by maintenance of myocardial energy and reduction in oxidative stress [17]. MCTs have been proposed for potential clinical application in the management of cardiac diseases in humans [18].

Inefficient mitochondrial energy function can contribute to oxidative stress via increased production of free radicals [19]. Alternative energy sources, such as MCTs, mitochondrial co-factors such as carnitine or carnitine precursors, plus antioxidants can help to address this by reducing production of free radicals and quenching those that are produced [12, 13, 20–22]. Taurine, a nutrient required for normal cardiac function, also serves as an antioxidant [20, 23]. Vitamin E, long known as a cellular antioxidant, also has anti-inflammatory properties [24–26]. Multiple studies have shown an inverse association between vitamin E intake and risk for cardiovascular disease in humans [26]. Long chain omega-3 fatty acids, especially eicosapentaenoic acid, help to reduce inflammatory mediators and oxidative stress, reduce cardiac arrhythmias, reduce cardiac remodeling and dysfunction, and reduce blood pressure [27, 28].

Magnesium (Mg) is a mineral critical for normal cardiac function and provides an antiarrhythmic action [29, 30]. It also reduces hypertension and provides antioxidant and anti-hyperlipidemic effects [31, 32]. In humans, inadequate Mg is correlated with heart failure and increased risk for various cardiovascular disorders [30, 33–35]. Mg deficiency contributes to mitral valve prolapse, and has been associated with increased calcification of mitral valves and intima media thickness in human patients with diabetes [36, 37].

Rather than attempting to modify one nutrient at a time, the diet tested in this study contained a cocktail of multiple supplemental nutrients (Table 1). Previous studies have shown that combinations of nutrients can perform better than single nutrient supplements [38, 39]. A blinded, randomized placebo-controlled interventional study was conducted, using echocardiogram evaluation as the primary endpoint, to evaluate the effect of the diet on progression of early stage MMVD in dogs. Clinical measures best associated with progression of mild MMVD include degree of mitral regurgitation (MR) and the echocardiographic variables left atrial diameter (LAD) and left atrial size measured as the ratio of left atrial to aortic root diameter (LA/Ao) [40, 41]. This study was performed in dogs naturally affected by MMVD. An age, sex and breed matched group of healthy dogs was included to assess any changes associated with this nutrient blend among healthy dogs.

**Table 1:** Composition of the control (CON) and cardiac protection blend (CPB) diets

Ingredients	CON		CPB	
	%Diet			
Basal diet mix^1^	90.70		86.00	
Egg Protein	1.99		0	
Beef fat	9.30		0	
MCT Oil	0		5.00	
Fish Oil	0		2.85	
Fish Meal	0		3.39	
L-lysine	0		1.26	
DL-methionine	0		1.02	
Taurine	0		0.13	
Magnesium sulfate	0		0.20	
DL alpha tocopherol, supplemental	0		0.15	
	%Dry matter		Per 100 Kcal ME	
Nutrient content^2^	CON	CPB	CON	CPB
			g/100 Kcal	
Crude protein	28.70	29.92	7.16	7.67
Fat (acid hydrolysis)	16.65	14.88	4.16	3.82
Crude fiber	3.82	3.92	0.95	1.01
Ash	5.49	5.92	1.37	1.52
Carbohydrate (by difference)	45.33	45.35	11.31	11.63
Lysine	1.16	2.12	0.29	0.54
Methionine	0.61	1.49	0.15	0.38
EPA and DHA	0.06	0.72	13.88	183.57
			mg/100 Kcal	
Na	0.23	0.24	58.55	61.46
Mg	0.11	0.14	28.02	35.20
Taurine	0.07	0.20	17.30	52.21
Vitamin E (α-tocopherol)^3^	0.15	0.84	3.80	21.46
ME (calc.), kcal/g	4.00	3.89		

ME, metabolizable energy; EPA, Eicosapentaenoic acid, and DHA, Decosahexaenoic acid, are omega-3 fatty acids from fish oil

^1^Basal diet composed on grains (corn, rice, wheat), proteins (poultry, corn gluten and corn germ meal), dietary fibers (beet pulp, cellulose), vitamins and minerals, and flavoring additives

^2^Average nutrient analysis is based on the mean of 3 separate manufacturing runs of diet

^3^Vitamin E level is expressed as either IU/g (in dry matter) or IU/100 kcal

## Results

Nineteen MMVD dogs (17 Beagles and 2 Miniature Schnauzers) and 17 age-, sex-, body condition-, and breed-matched healthy dogs (15 Beagles and 2 Miniature Schnauzers), were enrolled into the 6-month study. One MMVD dog in the CON diet group was removed from the study after the 3 month evaluation due to lymphoma. All other dogs remained in good health throughout the study. Five MMVD dogs were receiving enalapril, and 2 of them were also receiving pimobendan for at least 3 months prior to enrollment. These dogs were approximately equally divided among the 2 diet groups: the CPB group included one dog on enalapril only and one on both medications with the remaining 3 in the CON group. The medications were continued unchanged throughout the study.

No difference was found between the two diet groups in any of the echocardiogram or physical variables at baseline (Table 2). At baseline, all MMVD dogs were either at the ACVIM stage B1 (*N* = 15) or B2 (*N* = 4). The baseline mean for LA/Ao was 1.19 for MMVD dogs and 1.04 for healthy dogs (*P* = 0.008), while those of LAD were 2.01 cm and 1.90 cm, respectively (*P* = 0.38). The baseline serum chemistry and complete blood count values were all within normal reference ranges (data not shown). A *p*-value of 0.05 is considered statistically significant.

**Table 2:** Baseline values and characteristics of dogs

	MMVD		p-value	Healthy		p-value
	CON	CPB		CON	CPB	
Total number	9	10	nd	8	9	nd
Sex (M/F)	5/4	6/4	nd	4/4	5/4	nd
Age, years	11.2 (7.9–13.7)	10.5 (8.1–12.5)	0.45	9.9 (1.6–12.9)	10.3 (8.1–13.0)	0.79
Breed (Beagle/Miniature Schnauzer)	8/1	9/1	nd	7/1	8/1	nd
Physical examination variables						
Body weight, kg	10.4 (7.43–12.94)	9.8 (6.52–12.69)	0.55	11.0 (7.84–13.58)	12.0 (7.53–15.94)	0.35
BCS	5.2 (4–6)	5.0 (4–6)	0.48	5.8 (5–7)	5.6 (5–6)	0.55
Heart rate	118 (95–140)	112 (95–153)	0.57	120 (93–152)	120 (87–156)	0.99
SAP (mmHg)	190.8 (150–219)	171.0 (133–218)	0.10	171.2 (146–211)	191.9 (164–217)	0.04
DAP (mmHg)	104.9 (91–131)	106.2 (77–133)	0.84	99.5 (77–131)	114.3 (73–139)	0.11
Heart murmur grade (1/2/3/4)	0/4/4/1	1/3/5/1	0.46	0/0/0/0	0/0/0/0	nd
ACVIM stages (B1/B2)	7/2	8/2	nd	0/0	0/0	nd
Echocardiogram variables						
LAD (cm)	1.97 (1.47–2.58)	2.04 (1.39–2.67)	0.70	1.87 (1.46–2.3)	1.93 (1.37–2.26)	0.70
LA/Ao	1.16 (0.91–1.56)	1.22 (1.1–165)	0.54	1.00 (0.77–1.16)	1.07 (0.93–1.24)	0.28
LVD (cm)	3.11 (2.57–3.57)	3.17 (2.62–3.59)	0.69	2.92 (2.54–3.38)	3.00 (2.39–3.49)	0.64
MVR (m/s)	5.50 (5.03–6.28)	5.75 (5.40–6.32)	0.14	nd	nd	nd
EF (%)	74 (67–86)	70 (60–81)	0.14	70 (63–83)	68 (59–74)	0.50
MR (none or trace/mild/moderate/severe)	0/2/5/2	0/0/5/5	nd	7/1/0/0	8/1/0/0	nd

MMVD, myxomatous mitral valve disease; CON, control diet; CPB, cardiac protection blend; BCS, body condition score; LAD, left atrial diameter; LA/Ao, left atrial to aortic root ratio; LVD, left ventricular diameter; MVR, mitral regurgitation velocity; EF, ejection fraction; MR, mitral regurgitation; SAP, systolic arterial pressure; DAP, diastolic arterial pressure; ACVIM, American College of Veterinary Internal Medicine; nd, not detectable or determined. One MMVD dog in the control diet group developed lymphoma after three months and was subsequently removed from the study. Continuous variables are reported as mean (range). *P* values were calculated using t-test

### Results in healthy dogs

There were no significant changes in any measured parameters in the healthy dogs fed either diet over the 6-month study except BCS and SAP (Additional file 1: Table S1). Mean BCS started slightly, but not significantly (*P* = 0.14), lower at baseline in the CPB group and decreased slightly over the study, resulting in a mean BCS 0.7 units lower among dogs in the CPB group at 6 months compared with the CON group (*P* = 0.03). No BCS difference between 6 months and baseline was found within each group. Average SAP increased by 12.9 mmHg (*P* < 0.05) in the CON group over baseline while it decreased by 13.3 mmHg (*P* < 0.01) in the CPB group. There was no correlation between BCS and SAP (*P* = 0.40), nor between change in BCS and change in SAP (*P* = 0.65).

### Echocardiographic data in MMVD dogs

**Fig. 1:**
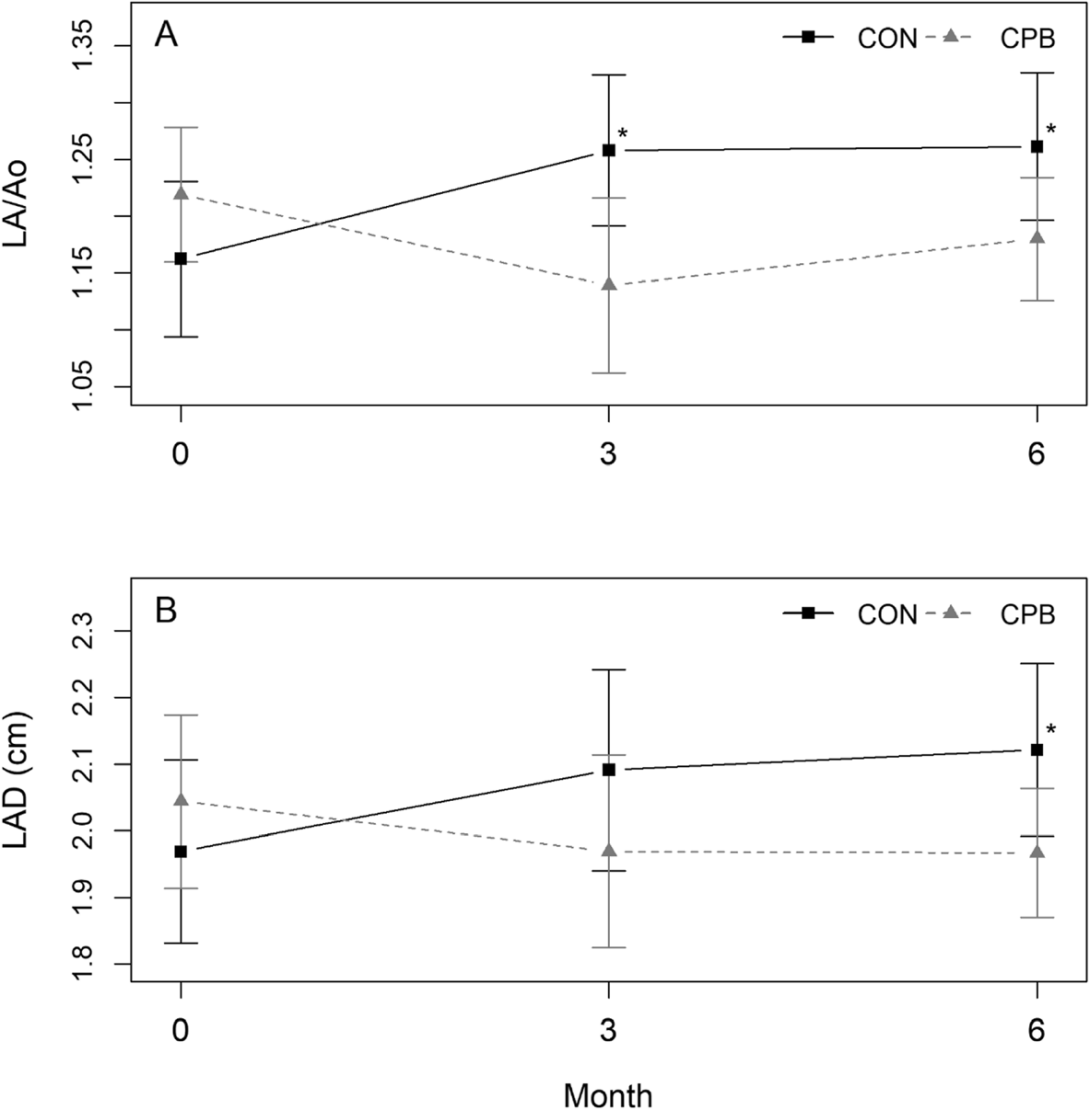
Effect of diet and time on left atrial size in MMVD dogs. Plots show means with standard error bars for (**a**) LA/Ao and (**b**) LAD in dogs with MMVD fed the control (CON) or test (CPB) diet. There was a significant diet by time interaction (*P* < 0.05) for both variables, with CON dog increasing and CPB dogs decreasing over time. *P < 0.05; ** *P* < 0.01

**Fig. 2:**
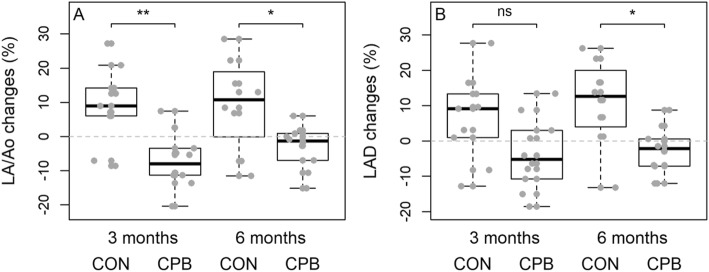
Percent change from baseline in (**a**) LA/Ao and (**b**) LAD at 3 months and 6 months in dogs with MMVD. The horizontal dashed line in each plot denotes baseline values. The bottom, middle, and top lines of the box represent 25th, 50th, and 75th percentiles. The whiskers indicate 1.5 times the interquartile range from the box. The *p*-values from the Student’s t-test are **(a)** 0.006 and 0.049 and **(b)** 0.054 and 0.025 respectively. “ns”, *P* ≥ 0.05, “*”, *P* < 0.05, “**”, *P* < 0.01. LA/Ao, left atrial to aortic root ratio; LAD, left atrial diameter; CON, control diet; CPB, cardiac protection blend

There was a significant interaction in diet by time in LA/Ao and LAD (*P* = 0.005 and *P* = 0.037, respectively) (Fig. 1, Additional file 1: Table S2). CON dogs showed a significant increase in LA/Ao over baseline at 3- and 6-months (*P* = 0.012 and *P* = 0.010, respectively) while the CPB dogs showed a trend of decreases (*P* > 0.05). For LAD, CON dogs showed increases over baseline at 3- and 6-months (*P* = 0.06, *P* = 0.022, respectively). No change over baseline was found in CPB dogs (P > 0.05). Further, we calculated the percent changes at 3 or 6 months over its baseline value for each dog. While no difference in LAD or LA/Ao was observed at baseline between diet groups, diet effect became evident by 3 months and continued through the 6-month study (LAD: *P* = 0.054 and *P* = 0.025; LA/Ao: *P* = 0.006, *P* = 0.049 respectively) (Fig. 2, Additional file 1: Table S3). In CON dogs, the average increases at 3 months and 6 months were 6.6 and 10.8% in LAD and 9.0 and 9.5% in LA/Ao, respectively. CPB dogs showed 3.7 and 2.9% reductions in LAD, and 7.1 and 2.9% reductions in LA/Ao, for the same 3 and 6-month times respectively. Six of the 10 CPB dogs showed decreases in LAD and LA/Ao after 6 months, compared with only 1 (LAD) or 2 (LA/Ao) of the CON dogs. No significant change was found in LV.

### Progression of MR and MMVD

**Fig. 3:**
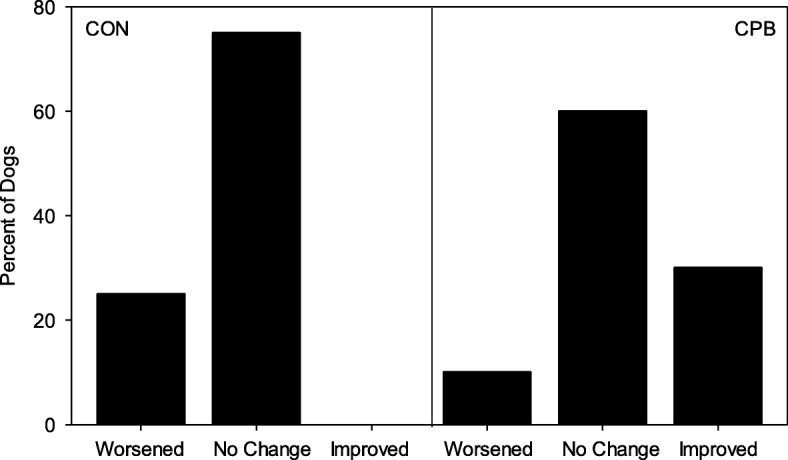
Effect of diet and time on severity of mitral regurgitation in MMVD dogs. Plots show percent of dogs that showed changes of at least one grade in mitral regurgitation after 6 months, compared with baseline, in dogs fed the control (CON) or the test (CPB) diet. *P* = 0.041 for diet by time interaction

**Fig. 4:**
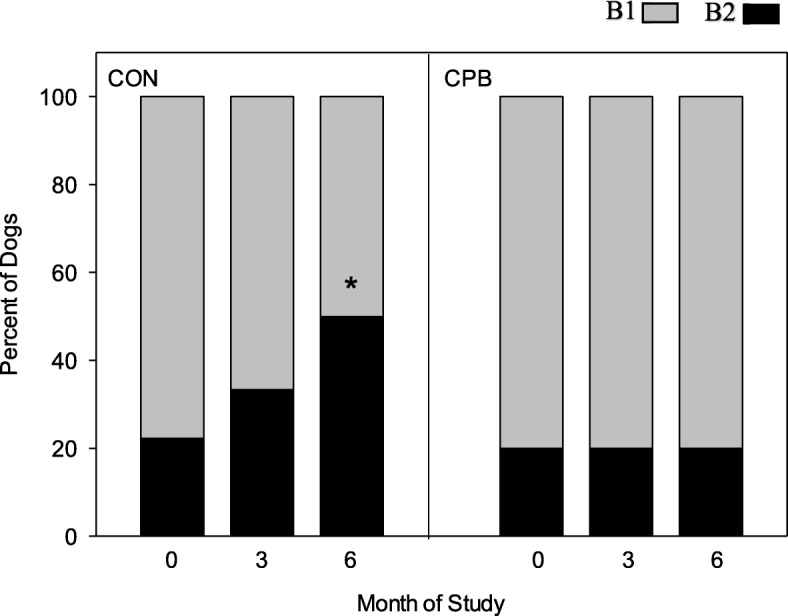
Progression of disease in MMVD dogs based on ACVIM stage. Figure shows percent of dogs in ACVIM Stage B1 or B2 at 0-, 3- and 6-months of the study. The diet by time interaction was significant at 3 and 6 months, *P* < 0.01. *CPB differed from CON at 6 months, *P* < 0.001

While the majority of CON dogs showed no change in grade (none/trace, mild, moderate, or severe) in the severity of MR, 2 (2/8) worsened by the end of the study and no CON dogs improved (Fig. 3, Additional file 1: Table S4): In contrast, just 1 (1/10) CPB dog progressed from moderate to severe, while 3 (3/10) improved in this group: 2 dogs improved from moderate to mild and 1 dog improved from severe to mild (P_diet x 6-month_ = 0.041). Consistent with this, CON dogs showed progression of MMVD from ACVIM stage B1 to B2 by 6 months, but none of the CPB dogs had progressed (Fig. 4, P_diet x 6-month_ = 0.001, Additional file 1: Table S4). Based on odd ratios, the likelihood for a CON dog being in B2 stage was 2 times that of a CPB dog at 3 months, increasing further to 4 times that of a CPB dog at 6 months.

### Correlation between LAD and BP in MMVD dogs

**Fig. 5:**
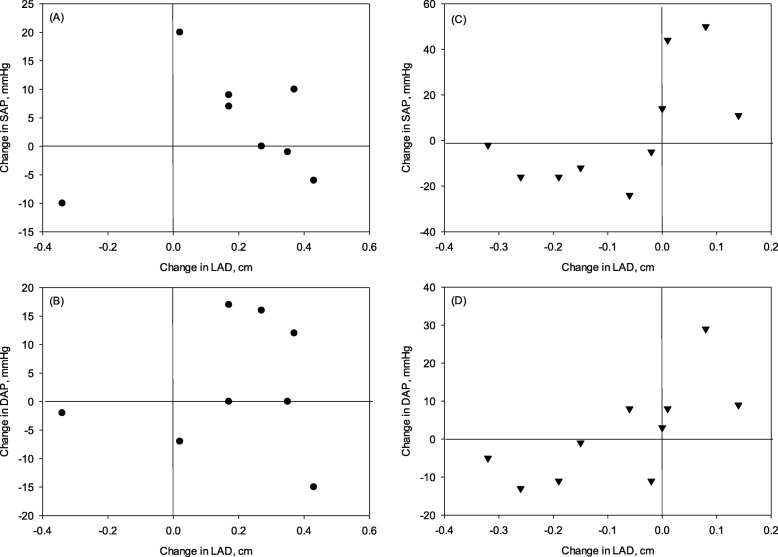
Correlation between changes in left arterial diameter (LAD) and blood pressure. Graphs show correlation between change in LAD and change in systolic arterial pressure (SAP) or diastolic arterial pressure (DAP), after 6 months on either (**a** and B) control (CON) or (C and D) test (CPB) diet. Correlations were significant for both SAP (r = 0.63, *P* = 0.05) and DAP (r = 0.67, *P* = 0.035) in CPB dogs, but no correlation was observed in CON dogs (*P* > 0.75)

Although blood pressure increased slightly over baseline in both diet groups by 6 months, these changes did not approach statistical significance. Within dog changes in LAD were positively correlated with changes in SAP and DAP in CPB dogs (r = 0.63 and 0.67; *P* = 0.050 and 0.035 respectively), but no correlation was observed in CON dogs (Fig. 5). Of interest was the observation that the same 6 CPB dogs that showed a reduction in LA enlargement also had a decrease in blood pressure.

## Discussion

To the best of our knowledge, this is the first dietary intervention to successfully delay progression of MMVD in dogs. The CPB was formulated to provide nutrients recognized to be of benefit for cardiac conditions, including antioxidants, carnitine precursors, taurine, magnesium and long-chain omega-3 fatty acids. Uniquely, it included MCTs as an alternative energy source. Prior metabolomic and transcriptomic data from our laboratory suggested that dogs with MMVD have compromised fat and glucose energy metabolism [14]. The MCT oil used provided a source of 8- and 10-carbon medium chain fatty acids. In contrast to long chain fatty acids, medium chain fatty acids do not require a carnitine-mediated transport pathway to cross the cell and mitochondrial membranes, thus can be readily absorbed and preferentially oxidized for ATP without increasing oxidative stress [18, 21, 42, 43]. Despite the potential benefits, only a limited number of studies have been conducted to explore MCTs in cardiac diseases, mostly in spontaneously hypertensive rat models. To our knowledge, no prior studies have investigated MCTs in cardiac diseases in dogs.

The MMVD CPB diet group showed improvement in left atrial enlargement as determined by echocardiography. Echocardiography provides the most commonly used noninvasive method for assessment of cardiac function in dogs. Left atrial enlargement represents the most reliable independent indicator for progression of cardiac disease in dogs with MMVD [41]. The risk of developing CHF increases with increasing left atrial size [44]. LA/Ao, normalized by aortic root diameter, provides a more consistent measurement of LAD for adult dogs and is body weight independent [44, 45]. Although MMVD is considered to be a slowly progressive condition, LA/Ao and LAD increased in MMVD-CON dogs by an average of 10% over baseline within this 6 month study. Importantly, MMVD-CPB dogs showed the opposite, with decreases in LA/Ao and LAD. Six (6/10) MMVD-CPB dogs had a reduction in both LA/Ao and LAD, and these same 6 dogs had a decrease in blood pressure. The changes in LA/Ao and LAD were evident within 3 months, and remained significant through 6 months. As expected, the baseline value of LA/Ao was significantly greater in MMVD dogs than in healthy dogs.

Progressive mitral regurgitation, which increases cardiac work, can lead to both atrial and ventricular remodeling and enlargement [1]. Although a similar trend was observed in LV reductions in both M-mode (3 and 6 months), and 2-D (3 months), the differences were not statistically significant. Most of the MMVD dogs in this study were in the early B1 stage. It is therefore possible that either the B1 dogs had normal LV, or they had small LV enlargement and the change was too small to be detected. However, the change in LA or LA/Ao was clear and consistent in our study. Our data suggest that at-risk dogs may have already experienced myocardial energy starvation prior to MMVD and that mitral regurgitation may lead to LA enlargement as early as B1 stage. It would be very interesting to test the hypothesis whether CPB can delay the onset of MMVD. .

Left atrial enlargement in MMVD reflects the degree of severity and progression of mitral valve regurgitation [46]. Consistent with the improvement in left atrial enlargement in CPB dogs, MR was reduced: 3/10 of CPB dogs had less MR compared with no improvement in CON dogs. In addition, 2/8 CON dogs showed an increase over baseline in MR compared with 1/10 CPB dogs. These changes also were reflected in ACVIM grading, with 3 CON dogs progressing from B1 to B2, compared to none in the CPB group. Taken together, our results demonstrated the efficacy of CPB in reducing left atrial size in dogs with early MMVD, and in slowing or reversing the progression of the disease at the preclinical stage.

Although it is recommended that BP be measured and monitored in dogs with MMVD, including asymptomatic patients [1], any reported association of BP change with MMVD progression in dogs has been scarce and lacking in consistency [47, 48]. The SAPs recorded in our study were higher than those reported previously [46–49], although no difference in SAP was found between MMVD and healthy dogs at baseline. We also noticed significant within-subject variations in BPs, which could be due to white coat syndrome, body movements or excitement of the animal during measurements. Despite this, changes in SAP and DAP over 6 months were significantly correlated with changes in LAD in the MMVD-CPB dogs. Remarkably, the same 6 dogs that had a reduction in LA enlargement also had a decrease in blood pressure. A recent longitudinal study of 5.5 million United Kingdom adults suggested association between elevated SAP and risk of mitral regurgitation [50]. Although no correlation was observed between changes in LAD and MR in our study, the correlation between LAD and SAP reported in our study raised interesting questions: Is elevated SAP associated with increased risk of MR in dogs? Is MR an inevitable consequence of canine ageing? Further studies with bigger cohorts of dogs are needed to address these questions.

We observed few differences over time in the healthy dogs. No adverse effects were observed. Of interest was the observation that SAP decreased in healthy dogs fed the CPB diet, a change which also occurred in some of the MMVD-CPB dogs. We also noticed significantly lower BCS at the end of the study in CPB dogs compared to CON dogs. However, the mean BCS in both groups was within the ideal range. The significance of this, if any, remains to be determined.

There were some limitations to the current study. One was the relatively small number of dogs in each group, with the majority of them being Beagles. More small dogs from different breeds should be enrolled in future studies. Differences in survival or time for the dogs to progress to B2 stage or develop CHF would be valuable end points, but this requires a very long-term study. In this study, we used LAD and LA/Ao as surrogate markers for MMVD progression. Secondly, five MMVD dogs in both treatment groups were on long-term medications. None of those dogs showed any improvement in MR. Among the five, three CON dogs had an increase in LA/Ao at 6 months (mean = 16.7%), one CPB dog had a 1.8% increase and the other one had a 2.7% decrease. Although those dogs were on these medications prior to the study and maintained them throughout, and the trend of changes in LA size in these dogs was similar to that in the larger groups, we are unable to rule out potential confounding effects from medications. Finally, factors such as direction of jet and left atrial pressure can influence the regurgitant jet appearance in the semi-quantitative assessment of mitral regurgitation using color flow Doppler. BP measures taken in this study used indirect oscillometric assessments. Although commonly used clinically, this method replies on the amplitude of cuff-pressure oscillations and is less accurate than the invasive direct measures of arterial pressure [51, 52]. Any movement by a dog may interfere with the ability of the device to provide an accurate estimate. A clinical study with more dogs is warranted to better assess the clinical utility of this diet.

## Conclusions

Our study demonstrated that dietary intervention with a blend of nutrients designed to address metabolic changes associated with MMVD in dogs was able to slow or reverse cardiac changes in dogs with early, preclinical MMVD. This study did not provide the opportunity to extrapolate effects from any single nutrient within the CPB. Rather, we believe the key nutrients acted synergistically to achieve the documented efficacy.

## Methods

### Study design

The study protocol was approved by the Animal Care and Use Committee of the Nestlé Purina PetCare Company and complied with all regulations set forth in the United States Department of Agriculture Animal Welfare Act. The study was a randomized, placebo-controlled trial, using a 2 by 2 factorial design to determine effects of diet, time and their interaction in dogs with MMVD or in age-, sex- and breed-matched healthy dogs. The study was performed over 6 months, with clinical measures collected at baseline, 3- and 6-months. The primary efficacy endpoint was the change in left atrial (LAD and LA/Ao) dimensions from baseline to 6 months between groups. The cardiologist was blinded to diet assignment throughout the study.

### Animals

Two groups of small and medium sized dogs living at a Nestlé Purina PetCare Center with body weight less than 15 kg were considered for inclusion in the study: those that had a cardiac left apical systolic murmur detectable on auscultation (MMVD group) and a group of sex-, age- and breed-matched healthy control dogs with no evidence of cardiac disease (healthy group). Randomization was performed using a random number generator in the statistical computing software. The algorithm consisted of 4 steps. 1. Two Schnauzers were randomly assigned either CON or CPB. 2. Male and female Beagles were randomly assigned either CON or CPB. 3. Student’s t-test was performed on age, body weight, and murmur grade (for DMVD group). 4. If no difference was found in step 3, randomization was completed. Otherwise, repeated steps 1–4.

All dogs were subsequently re-evaluated via echocardiography performed by a board-certified veterinary cardiologist (AH). All MMVD dogs were classified as either stage B1 or B2, using the ACVIM guidelines for diagnosis of MMVD [1]. Dogs on cardiac medications prior to enrollment were maintained on the medications throughout the study. Healthy dogs were determined to be healthy based on normal physical examination, serum biochemistry profiles, and echocardiogram. Dogs within each cardiac health group were further divided into 2 groups randomized by age, sex, breed, body weight and murmur grade (for the MMVD group), then randomly assigned to be fed one of the two study diets for 6 months.

Dogs were housed individually in adjoining indoor-outdoor runs with natural and additional lighting on a 12 h cycle. Open access was provided between adjoining runs except during feeding. In addition, all dogs were provided regular exercise such as walks and time in play yards, and daily socialization with other dogs and caretakers. Dogs were fed their assigned diets as their sole source of nutrition for 6 months: water was available ad libitum. Clinical measures were taken at baseline, 3 and 6 months. After the study, dogs remained in the Nestlé Purina PetCare Center where they continued to receive full wellbeing and veterinary cares from the pet care staffs and veterinarians.

### Diets

The study diets were formulated to be isocaloric and isonitrogenous, and to provide complete and balanced nutrition for adult dogs (Nestlé Purina PetCare Company, St. Louis, MO). One diet contained a cardiac protection blend (CPB) of nutrients that included MCTs; long-chain omega-3 fatty acids; lysine and methionine (carnitine precursors); vitamin E (an antioxidant); magnesium; and taurine (Table 1). The fat provided to the CPB diet from MCTs and fish oil was balanced by exchange for beef fat in the control diet (CON). Dogs were individually fed once daily to maintain stable body weight throughout the study [53]. No treats or other supplements were allowed during the course of study.

### Clinical measurements

The primary outcomes of this study were measures to assess changes in progression of MMVD, specifically degree of mitral regurgitation (MR) and the echocardiographic variables left atrial diameter (LAD) and left atrial size measured as the ratio of left atrial to aortic root diameter (LA/Ao).

All clinical examinations, including physical examinations, echocardiograms, and indirect blood pressure, were performed without sedation in a quiet examination room at baseline, 3 months, and 6 months. Dogs were presented for clinical evaluations in random orders. BCS was evaluated using a 9-point scale where a score of 4 or 5 is considered ideal [54]. All cardiac evaluations, including cardiac auscultation, standard 2-dimensional (2-D), M-mode, and color flow Doppler echocardiography, were performed by the same veterinary cardiologist who was blinded to the diet assignments of the dogs. The evaluations were performed using a Mylab Alpha ultrasonographic unit equipped with a 1–4 MHz transducer, and ECG monitoring. Both LA/Ao and LAD were measured in 2-D. Left atrial to aortic root ratio (LA/Ao) was measured as the ratio of maximal left-atrial dimension, which was measured using the left atrium short-axis diameter, and the internal diameter of the aorta measured by extending a line from the convex curvature of the wall of the right aortic sinus to the opposite wall of the aorta lining up with the junction of non-coronary and left coronary aortic valve cusps on the first frame after aortic valve closure [55]. Left atrial diameter (LAD) was measured by extending the same line to the distant margin of the left atrium [44]. 2-D measurements of left ventricle (LV) were performed according to the standard protocol [56]. Echo measurements were made offline after patient visits by the same cardiologist (AH) who was blinded at the time of measurement. The intensity of left apical systolic murmur was graded using a 1 to 6 scale, where grade 1 is the softest audible in a quiet exam room with particular effort, and grade 6 is the loudest with a palpable thrill audible with the stethoscope just removed from the patient’s chest [57]. Mitral regurgitation (MR) severity was estimated using receiving chamber analysis by calculating the maximal ratio of the regurgitant jet area to left atrial area using the color flow Doppler mode, as described previously [58, 59]: MR was graded as none or trace if the ratio was 0–5%, mild if the ratio was less than 20%, moderate if between 20 and 50%, or severe if greater than 50%. Mitral regurgitation velocity (MRV) was measured from the left parasternal apical four chamber view using continuous wave Doppler. Blood pressure (BP), including systolic arterial pressure (SAP), diastolic arterial pressure (DAP), and mean arterial pressure (MAP), and heart rate (HR) were measured using an automated oscillometric device, petMAP graphic (Ramsey Medical, Inc., Tampa, FL). Dogs were placed on an exam table in a standing position where the cuff was placed on the left forearm. The median of 3 consecutive measurements was calculated.

Fasting venous blood samples were collected at baseline, 3- and 6-months for serum chemical profile analysis and complete blood counts. Complete blood counts were performed using Sysmex xs-1000i Hematology Analyzer (Lincolnshire, IL). Serum biochemistry profiles were analyzed using Roche Cobas c311 Chemistry Analyzer (Indianapolis, IN).

### Statistical data analysis

As this study was the first of its kind, no prior information was available to use for a power analyses and dogs were recruited based on availability of canines with MMVD. Data were treated as continuous outcomes except mitral regurgitation severity, ACVIM stage, and murmur grade, which were considered ordinal outcomes. Measures were taken for each dog at 3 time points, so mixed model analyses were used to account for repeated measures. Independent t tests were used to compare baseline values between healthy and MMVD dogs.

Continuous variables were analyzed using a linear mixed model with the “lme4” package in R [60]. The model included diet, time, and an interaction of diet by time as fixed effects, adjusted for sex, breed, and body weight. Dog was considered as a random effect to account for repeated measures. Residuals were used to check model assumptions. The type III sums of squares were used to test overall significance of a variable. Model estimated parameters were used to assess changes from baseline for each group at each time point. Post hoc independent t tests were used to compare groups at each time point with a Bonferroni adjustment for multiple testing using the R package “lsmeans” [61]. A *p*-value less than 0.05/3 = 0.017 was considered significant.

Ordinal variables were analyzed using the cumulative link mixed model with the “ordinal” package in R [62]. The model included diet, time, and an interaction of diet by time as fixed effects. Dog was considered as a random effect to account for repeated measures. Residuals were used to check model assumptions. Model estimated parameters were used to assess changes from baseline for each group at each time point. Post hoc tests on a latent variable with a set of cut points were used to compare differences between groups at each time point, with a Bonferroni adjustment for multiple testing [61]. A p-value less than 0.05/3 = 0.017 was considered significant.

Pearson correlations were performed to evaluate linear associations between 6-month changes in left atrial size and those in blood pressures within each diet group. Odds ratios were calculated for changes from baseline in ACVIM stage at 3- and 6-months.

## Supplementary information

**Additional file 1: Table S1.** Systolic arterial pressure and body condition score in healthy dogs. **Table S2.** Left atrial size at baseline, 3 months and 6 months in MMVD dogs. **Table S3.** Percent changes in left atrial size over baseline in MMVD dogs. **Table S4.** Murmur grade, mitral regurgitation severity and ACVIM stage.

## Data Availability

The datasets used and/or analyzed during the current study are available from the corresponding author on reasonable request.

## Abbreviations

ACVIM: American College of Veterinary Internal Medicine
BP: Blood pressure
CHF: Congestive heart failure
CON: Control diet
CPB: Cardiac protection blend
DAP: Diastolic arterial pressure
HR: Heart rate
LA: Left atrium
LA/Ao: Left atrial to aortic root diameter ratio
LV: Left ventricle
MAP: Mean arterial pressure
MCT: Medium chain triglycerides
MMVD: Myxomatous mitral valve disease
MR: Mitral regurgitation
SAP: systolic arterial pressure
