# Supplementary material for: Dietary intervention reduces left atrial enlargement in dogs with early preclinical myxomatous mitral valve disease: a blinded randomized controlled study in 36 dogs
Source: BMC Vet Res. 2019 Nov 27;15:425. doi: 10.1186/s12917-019-2169-1 (PMC6882217; doi:10.1186/s12917-019-2169-1)
Supplement: Supplementary file 1 — Additional file 1: Table S1. Systolic arterial pressure and body condition score in healthy dogs. Table S2. Left atrial size at baseline, 3 months and 6 months in MMVD dogs. Table S3. Percent changes in left atrial size over baseline in MMVD dogs. Table S4. Murmur grade, mitral regurgitation severity and ACVIM stage. [file 12917_2019_2169_MOESM1_ESM.docx]

| **Table S1**: Systolic arterial pressure and body condition score in healthy dogs | | | | | | | | |
| --- | --- | --- | --- | --- | --- | --- | --- | --- |
|  |  | Mean (SD) | | | ANOVA p-values | | |  |
|  |  | Month 0 | Month 3 | Month 6 | Diet | Time | Diet x Time |  |
| SAP | CON | 171.25 (20.78) | 174.25 (21.67) | 184.38 (21.65) | 0.4168 | 0.7294 | 0.0063 |  |
|  | CPB | 191.89 (17.13) | 194.56 (24.85) | 178.67 (24.02) |  |  |  |  |
| BCS | CON | 5.75 (0.71) | 5.38 (0.52) | 5.88 (0.64) | 0.0345 | 0.1007 | 0.4356 |  |
|  | CPB | 5.56 (0.53) | 5.22 (0.44) | 5.33 (0.5) |  |  |  |  |

| **Table S2**: Left atrial size at baseline, 3 months and 6 months in MMVD dogs | | | | | | | |
| --- | --- | --- | --- | --- | --- | --- | --- |
|  |  | Mean (SD) | | | ANOVA  p-values | Pairwise  p-values | |
|  |  | Month 0 | Month 3 | Month 6 | Diet x Time | 0 v. 3 | 0 v. 6 |
| LA/Ao | CON | 1.16 (0.21) | 1.26 (0.20) | 1.26 (0.18) | 0.0047 | 0.012 | 0.010 |
|  | CPB | 1.22 (0.19) | 1.14 (0.24) | 1.18 (0.17) |  | 0.06 | 0.26 |
| LAD | CON | 1.97 (0.41) | 2.09 (0.45) | 2.12 (0.37) | 0.0367 | 0.06 | 0.022 |
|  | CPB | 2.04 (0.41) | 1.97 (0.46) | 1.97 (0.31) |  | 0.45 | 0.24 |

| **Table S3**: Percent changes in left atrial size over baseline in MMVD dogs | | | | | |
| --- | --- | --- | --- | --- | --- |
|  |  | Mean (SD) | | P values  (t-test) | |
|  |  | Month 3 | Month 6 | Month 3  CPB v. CON | Month 6  CPB v. CON |
| LA/Ao % Change From Baseline | CON | 9.04 (11.68) | 9.52 (13.65) | 0.006 | 0.049 |
|  | CPB | -7.08 (8.12) | -2.93 (6.30) |  |  |
| LAD % Change From Baseline | CON | 6.59 (12.44) | 10.77 (12.65) | 0.054 | 0.025 |
|  | CPB | -3.67 (10.20) | -2.87 (6.74) |  |  |

| **Table S4:** Murmur grade, mitral regurgitation severity and ACVIM stage | | | | | | | | | | | | | |
| --- | --- | --- | --- | --- | --- | --- | --- | --- | --- | --- | --- | --- | --- |
|  |  | Murmur Grade | | | | MR | | | ACVIM | | | |  |
|  |  | 1 | 2 | 3 | 4 | Mild | Mod | Sev | B1 | | B2 | |  |
| Month 0 | CON | 0 | 4 | 4 | 1 | 2 | 5 | 2 | 7 | 2 | |  |  |
|  | CPB | 1 | 3 | 5 | 1 | 0 | 5 | 5 | 8 | | 2 | |  |
| Month 3 | CON | 0 | 4 | 4 | 1 | 2 | 4 | 3 | 6 | | 3 | |  |
|  | CPB | 1 | 3 | 5 | 1 | 0 | 4 | 6 | 8 | | 2 | |  |
| Month 6 | CON | 0 | 4 | 4 | 1 | 1 | 4 | 3 | 4 | | 4 | |  |
|  | CPB | 1 | 3 | 5 | 1 | 3 | 2 | 5 | 8 | | 2 | |  |
